# Supplementary material for: Caspase-1-dependent spatiality in triple-negative breast cancer and response to immunotherapy
Source: Nat Commun. 2024 Oct 1;15:8514. doi: 10.1038/s41467-024-52553-6 (PMC11445480; doi:10.1038/s41467-024-52553-6)
Supplement: Supplementary file 1 — Supplementary Information [file 41467_2024_52553_MOESM1_ESM.pdf]

# Caspase-1-dependent spatiality in triple-negative breast cancer and response to immunotherapy

**Authors:** Weiyue Zheng[1,‡], Wanda Marini[1,‡], Kiichi Murakami[1], Valentin Sotov[1], Marcus Butler[1,2,3], Chiara Gorrini[1,4], Pamela S. Ohashi[1,5,6] and Michael Reedijk[1,6,7,*]

## Affiliations:

[1]Princess Margaret Cancer Centre, University Health Network, Toronto, Ontario, Canada.

[2]Department of Medical Oncology and Hematology, Princess Margaret Cancer Centre, University Health Network, Toronto, Ontario, Canada.

[3]Department of Medicine, Division of Medical Oncology, University of Toronto, Toronto, Ontario, Canada.

[4]School of Molecular and Cellular Biology, University of Leeds, Leeds, UK.

[5]Department of Immunology, University of Toronto, Toronto, Ontario, Canada.

[6]Department of Medical Biophysics, University of Toronto, Toronto, Ontario, Canada.

[7]Department of Surgical Oncology, Princess Margaret Cancer Centre, University Health Network, Toronto, Ontario, Canada.

‡ These authors contributed equally

* Corresponding author. Email: Michael.Reedijk@uhn.ca

**Supplementary information**

Supplementary Figure 1-8

Supplementary Table 1-3

Supplementary References 1-4

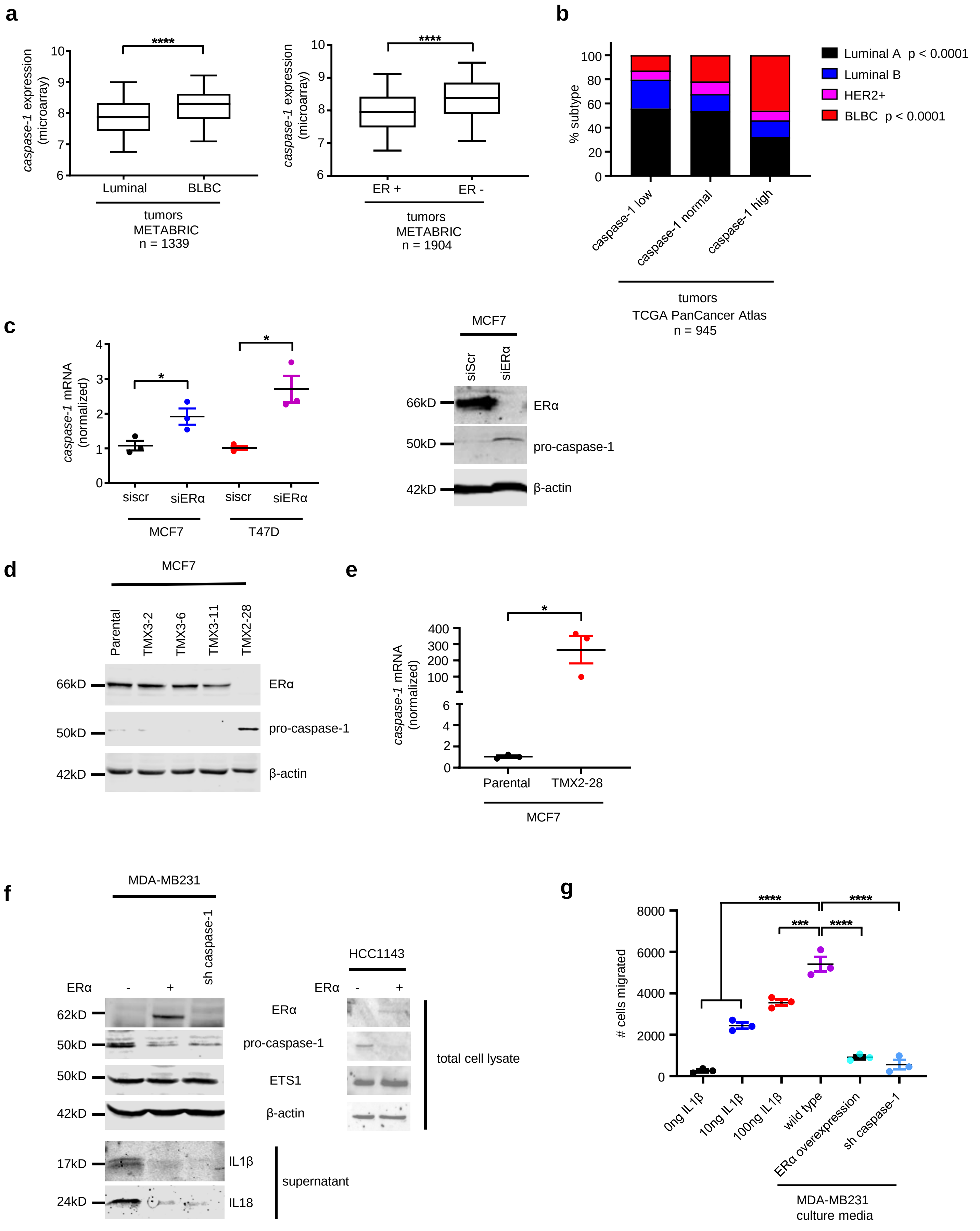

a
caspase-1 expression (microarray)
****
Luminal
BLBC
tumors
METABRIC
n = 1339
ER +
ER -
tumors
METABRIC
n = 1904
b
% subtype
Luminal A p < 0.0001
Luminal B
HER2+
BLBC p < 0.0001
caspase-1 low
caspase-1 normal
caspase-1 high
tumors
TCGA PanCancer Atlas
n = 945
c
caspase-1 mRNA (normalized)
siscr
siERα
MCF7
T47D
MCF7
siScr
siERα
66kD
ERα
50kD
pro-caspase-1
42kD
β-actin
d
MCF7
Parental
TMX3-2
TMX3-6
TMX3-11
TMX2-28
66kD
ERα
50kD
pro-caspase-1
42kD
β-actin
e
caspase-1 mRNA (normalized)
Parental
TMX2-28
MCF7
f
MDA-MB231
sh caspase-1
ERα
62kD
ERα
50kD
pro-caspase-1
50kD
ETS1
42kD
β-actin
17kD
IL1β
24kD
IL18
supernatant
HCC1143
total cell lysate
g
# cells migrated
0ng IL1β
10ng IL1β
100ng IL1β
wild type
ERα overexpression
sh caspase-1
MDA-MB231
culture media

**Fig. S1. Caspase-1 expression is ERα-dependant. a** mRNA expression of caspase-1 in human breast tumor samples (METABRIC dataset[1,2]) grouped either by subtype (1140 luminal and 199 BLBC samples) or by ERα expression (1459 ER+ and 445 ER- samples) (mean with 95% CIs, two-tailed, unpaired t-test with equal variances **** : $p < 0.0001$). **b** Proportion of breast cancer subtypes in caspase-1 low, normal, and high subgroups of human breast cancer (TCGA PanCancer Atlas dataset[1,2]) divided by a cutoff z-score of +/-1. P-values represent the significance level of a positive association between caspase-1 high tumors and the basal-like subtype, and a negative association between caspase-1 high tumors and the luminal A subtype (Fisher's exact test). **c** Caspase-1 mRNA level in ER positive MCF7 and T47D cell lines with siRNA transient KD of ERα, quantified by real time quantitative polymerase chain reaction (RTqPCR) (n = 3 biologically independent experiments, mean with SEM, two-tailed, unpaired t-test with equal variances, MCF7 siscr vs siERα: * : $p = 0.0367$; T47D siscr vs siERα: * : $p = 0.0125$) and associated immunoblot of pro-caspase-1 protein in the MCF7 cells . β-actin is included as a loading control. **d** Immunoblot of ERα and pro-caspase-1 in parental MCF7 and several tamoxifen-resistant MCF7 cell lines. **e** Caspase-1 mRNA levels in tamoxifen-resistant TMX2-28 cells compared to parental MCF7 (n = 3 independent biologically replicates, mean with SEM, two-tailed, unpaired t-test with equal variances, * : $p = 0.0351$). **f** Immunoblot of ERα, pro-caspase-1, and ETS1 from wild type MDA-MB231 or HCC1143 TNBC cells, cells overexpressing ERα, and cells with shRNA KD of caspase-1 and associated immunoblot of IL1β and IL18 in MDA-MB231 cell culture supernatant. **g** Transwell migration assay of THP-1 macrophages after the addition of increasing concentrations of recombinant IL1β, or conditioned media from wild type MDA-MB231 cells, cells overexpressing ERα, and cells with shRNA KD of caspase-1 (n = 3 biologically independent experiments, mean with SEM, one-way ANOVA with Dunnett's multiple comparisons test, *** : $p = 0.0003$, **** : $p < 0.0001$). n = 3 biologically independent experiments for immunoblots and representative images are shown. Source data are provided as a Source Data file.

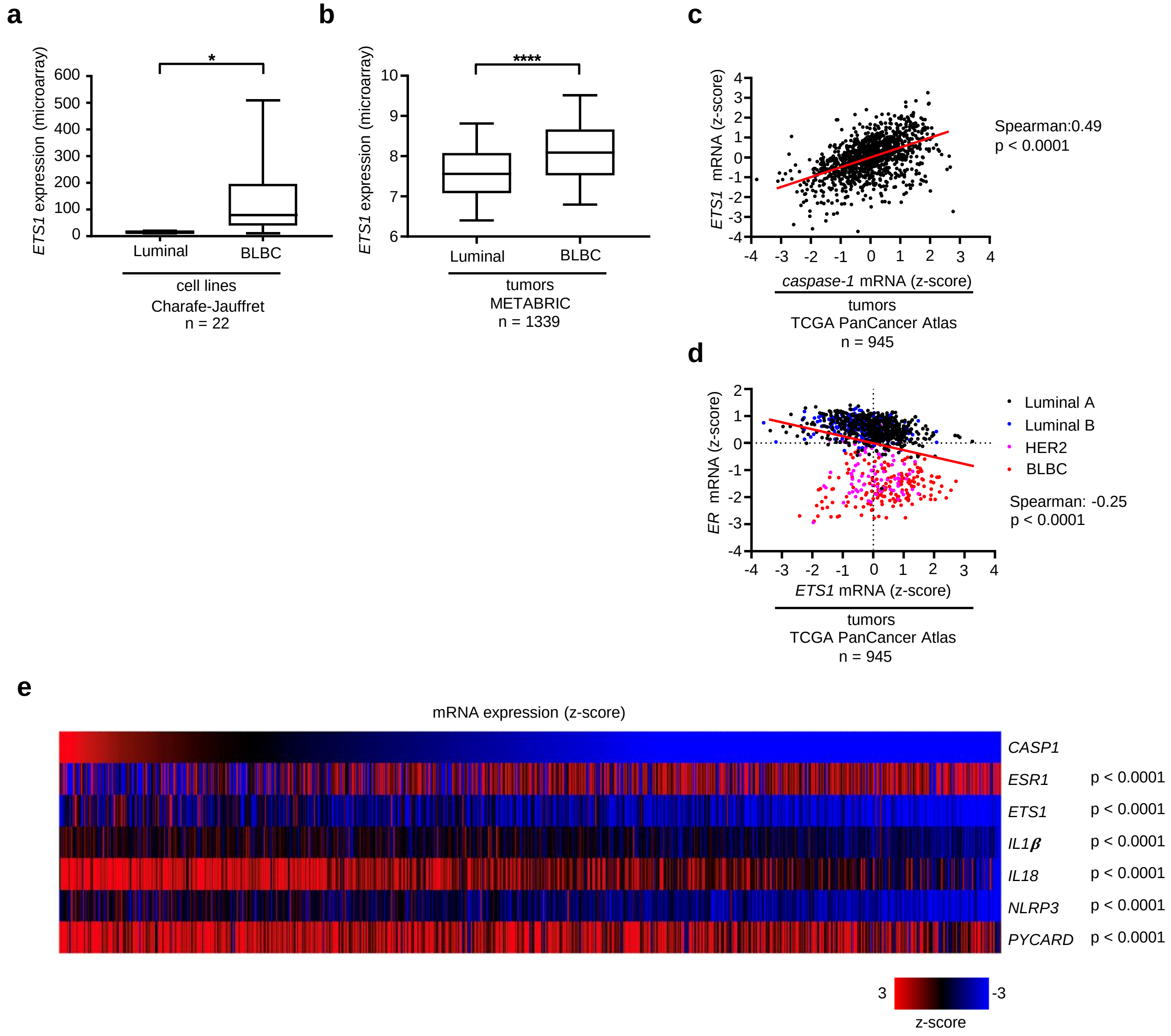
a
ETS1 expression (microarray)
600
500
400
300
200
100
0
*
Luminal
BLBC
cell lines
Charafe-Jauffret
n = 22
b
ETS1 expression (microarray)
10
9
8
7
6
****
Luminal
BLBC
tumors
METABRIC
n = 1339
c
ETS1 mRNA (z-score)
caspase-1 mRNA (z-score)
Spearman:0.49
p < 0.0001
tumors
TCGA PanCancer Atlas
n = 945
d
ER mRNA (z-score)
ETS1 mRNA (z-score)
Luminal A
Luminal B
HER2
BLBC
Spearman: -0.25
p < 0.0001
tumors
TCGA PanCancer Atlas
n = 945
e
mRNA expression (z-score)
CASP1
ESR1 p < 0.0001
ETS1 p < 0.0001
IL1β p < 0.0001
IL18 p < 0.0001
NLRP3 p < 0.0001
PYCARD p < 0.0001
3
-3
z-score

**Fig. S2. ETS1 is associated with breast cancer subtype and caspase-1 expression. a** mRNA expression level of ETS1 in 22 human breast cancer cell lines (13 Luminal and 9 BLBC cell lines) (Charafe-Jauffret dataset[3]), quantified by microarray, classified into two groups based on subtype (luminal vs BLBC), mean 95% CI, two-tailed, unpaired Welch's t-test with unequal variances, * : $p = 0.0376$). **b** mRNA expression level of ETS1 in 1339 human breast cancer samples (1140 luminal and 199 BLBC samples) (METABRIC dataset[1,2]) quantified by microarray, classified into two groups based on subtype (luminal vs BLBC) (mean 95% CI, two-tailed, unpaired t-test with equal variances, **** : $p < 0.0001$). Association between ETS1 and caspase-1 mRNA expression (**c**), and between ETS1 and ER mRNA expression (**d**) in 945 human breast cancer samples (TCGA PanCancer Atlas dataset[1,2]) quantified by RNAseq. **e** Heatmap expression levels of caspase-1 (*CASP1*), ER (*ESR1*), *ETS1, IL1β, IL18*, and inflammasome components *NLRP3* and ASC (*PYCARD*) in human breast tumors from the TCGA PanCancer Atlas dataset[1,2]. P-values represent positive Spearman correlations between caspase-1 and other listed genes, with the exception of *ER* (negative Spearman correlation). Source data are provided as a Source Data file.

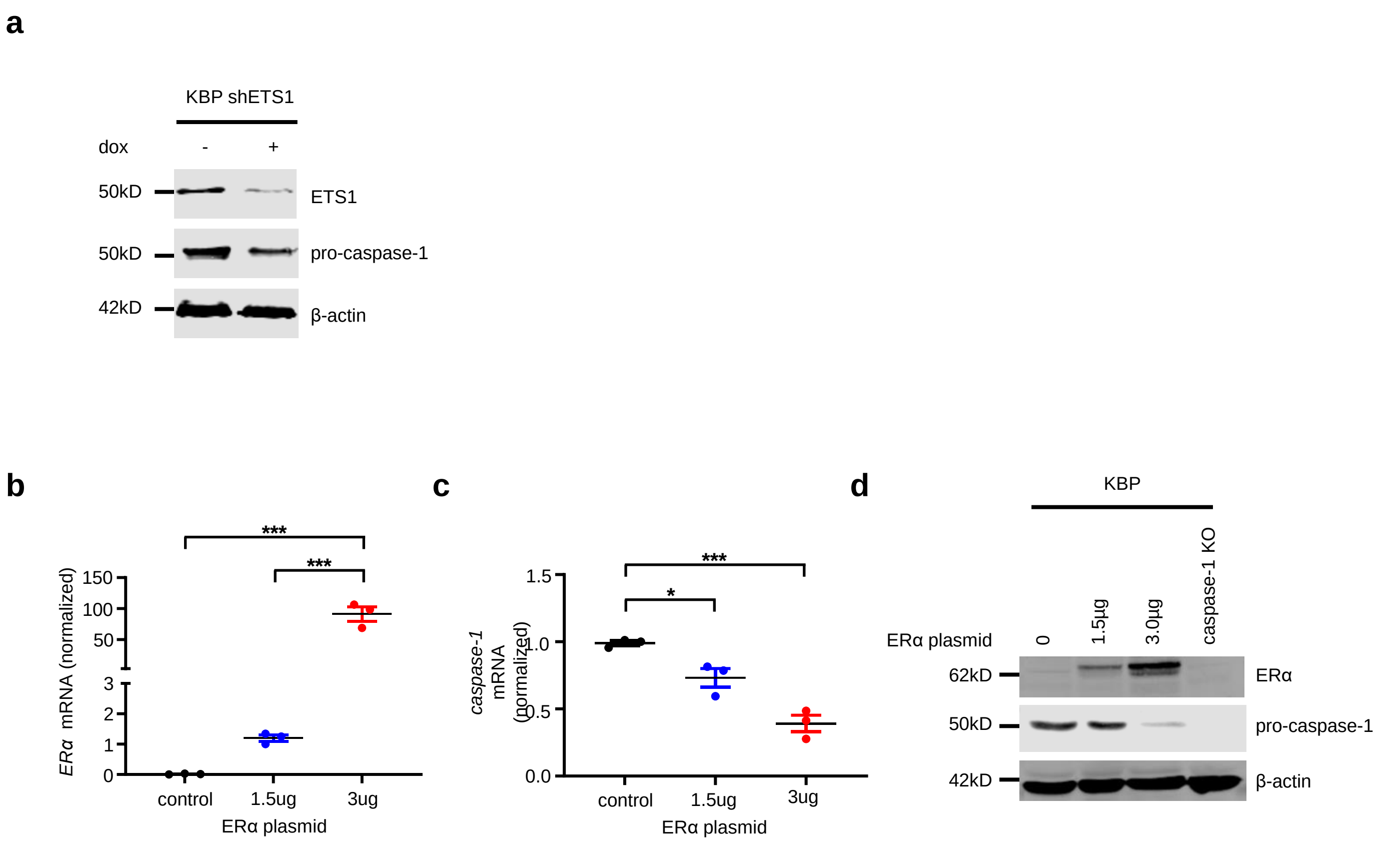
a
KBP shETS1
dox
-
+
50kD
ETS1
50kD
pro-caspase-1
42kD
β-actin
b
***
***
150
100
50
3
2
1
0
ERα mRNA (normalized)
control
1.5ug
3ug
ERα plasmid
c
***
*
1.5
1.0
0.5
0.0
caspase-1 mRNA (normalized)
control
1.5ug
3ug
ERα plasmid
d
KBP
ERα plasmid
0
1.5µg
3.0µg
caspase-1 KO
62kD
ERα
50kD
pro-caspase-1
42kD
β-actin

**Fig. S3. ERα and caspase-1 are inversely related in murine TNBC**. **a** Immunoblot of ETS1 and pro-caspase-1 in lysates from mouse KBP TNBC cells containing a doxycycline-inducible shRNA targeting ETS1, treated without (-) and with (+) doxycycline (dox). ERα (control vs 3µg: *** : p = 0.0002; 1.5µ vs 3µg: *** : p = 0.0002) (**b**) and caspase-1 (* : p = 0.034; *** : p = 0.006) (**c**) mRNA levels in KBP cells transiently transfected with serial amounts (0µg, 1.5µg, 3µg) of a mouse ERα-expressing plasmid. **d** Immunoblot of ERα and pro-caspase-1 in KBP cells transiently transfected with ERα-expressing plasmid (n = 3 biologically independent experiments, including immunoblots where representative images are shown; mean with SEM, one-way ANOVA with Dunnett's multiple comparisons test). Source data are provided as a Source Data file.

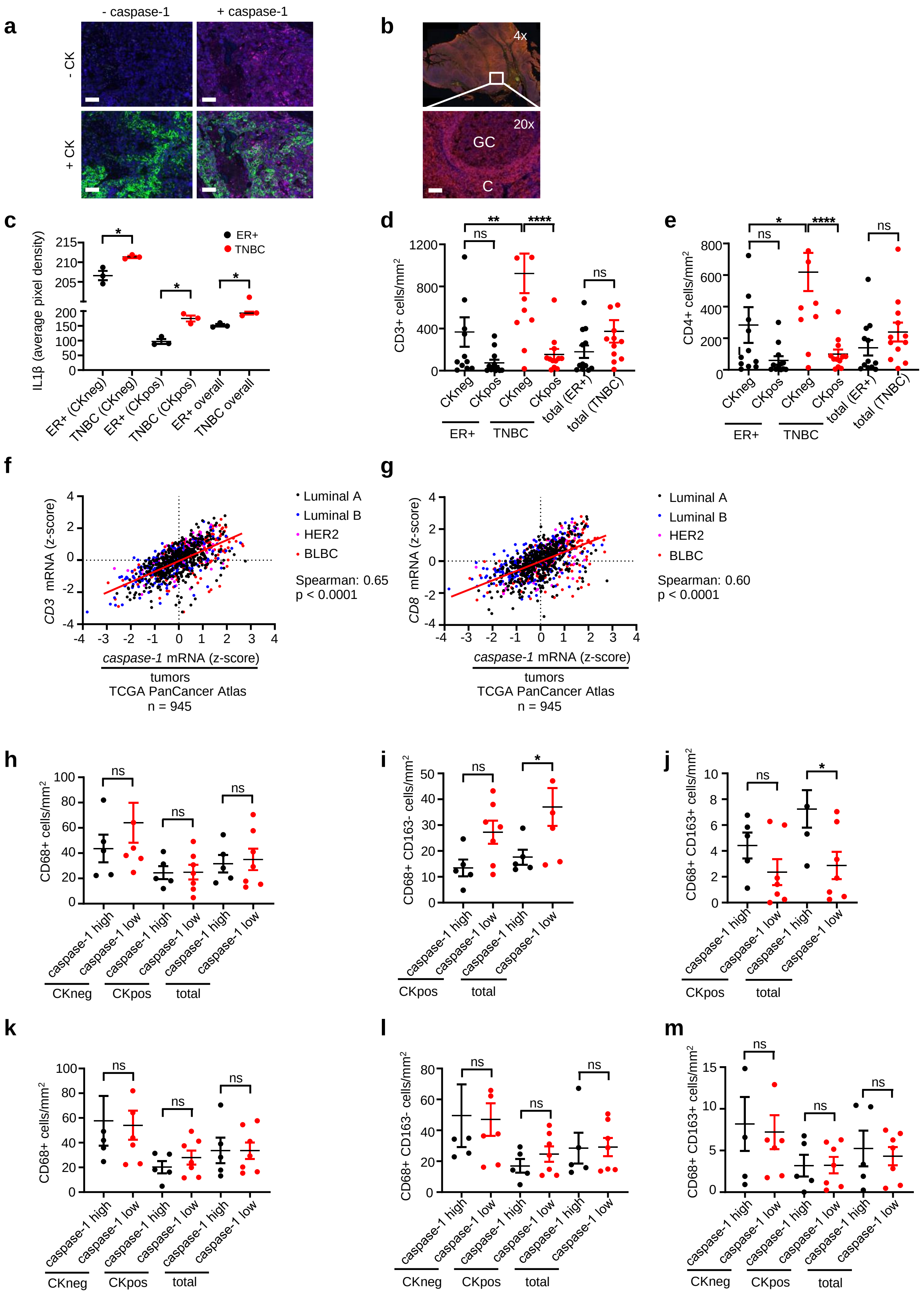
a
- caption-1
+ caspase-1
- CK
+ CK
b
4x
20x
GC
C
c
IL1β (average pixel density)
ER+
TNBC
ER+ (CKneg)
TNBC (CKneg)
ER+ (CKpos)
TNBC (CKpos)
ER+ overall
TNBC overall
d
CD3+ cells/mm²
e
CD4+ cells/mm²
CKneg
CKpos
total (ER+)
total (TNBC)
ER+
TNBC
ns
f
CD3 mRNA (z-score)
caspase-1 mRNA (z-score)
tumors
TCGA PanCancer Atlas
n = 945
Luminal A
Luminal B
HER2
BLBC
Spearman: 0.65
p < 0.0001
g
CD8 mRNA (z-score)
Spearman: 0.60
p < 0.0001
h
CD68+ cells/mm²
caspase-1 high
caspase-1 low
CKneg
CKpos
total
i
CD68+ CD163- cells/mm²
j
CD68+ CD163+ cells/mm²
k
l
m

**Fig. S4. Caspase-1 is associated with spatial immunophenotype in breast cancer. a** Multiplex IHC of TNBC probed with secondary antibody only (negative control, -caspase-1) or with anti-caspase-1 and secondary antibodies (+caspase-1) (DAPI: blue, CK: green, caspase-1: magenta; scalebar corresponds to 100$\mu$m). **b** IHC of human palatine tonsil (4X) with high magnification view of a lymph nodule (20X, scalebar corresponds to 100$\mu$m) confirming absence of germinal center (GC) staining and positive staining of the corona (C)[4]. **c** Multiplex IHC quantification of IL1$\beta$ in the stromal compartment (CKneg), intratumoral compartment (CKpos), or both (overall) in ER+ and TNBC tumor samples (n = 3 ER+, n = 3 TNBC; ER+ (CKneg) vs TNBC (CKneg) * : p = 0.0181; ER+ (CKpos) vs TNBC (CKpos) * : p = 0.0275; ER+ overall vs TNBC overall * : p = 0.0273) . Multiplex IHC quantification of CD3+ lymphocytes (** : p = 0.009; **** : p < 0.0001) (**d**) and CD4+ T-cells (* : p = 0.0315; **** : p < 0.0001) (**e**) in ER positive (ER+) and TNBC, in the stromal (CKneg) or intratumoral (CKpos) compartments, or overall (total) (n = 12 ER+, n = 12 TNBC). Association between CD3 and caspase-1 mRNA expression (**f**), and between CD8 and caspase-1 mRNA expression (**g**) in 945 human breast cancer samples (TCGA PanCancer Atlas dataset[1,2]) quantified by RNAseq. Quantification of CD68+ macrophages (n = 7) (**h, k**), CD68+ CD163- M1-like macrophages (* : p = 0.0286, n = 7) (**i, l**) and CD68+ CD163+ M2-like macrophages (* : p = 0.0311, n = 7) (**j, m**) within the CKneg and CKpos compartments, or overall (total). Caspase-1 high and low groups were categorized according to intratumoral (**h** - **j**) or stromal (**k** - **m**) caspase-1 quantification, divided by the mean caspase-1 value in those compartments. Mean with SEM, one-way ANOVA with Bonferroni's multiple comparisons test. ns = not significant. Source data are provided as a Source Data file.

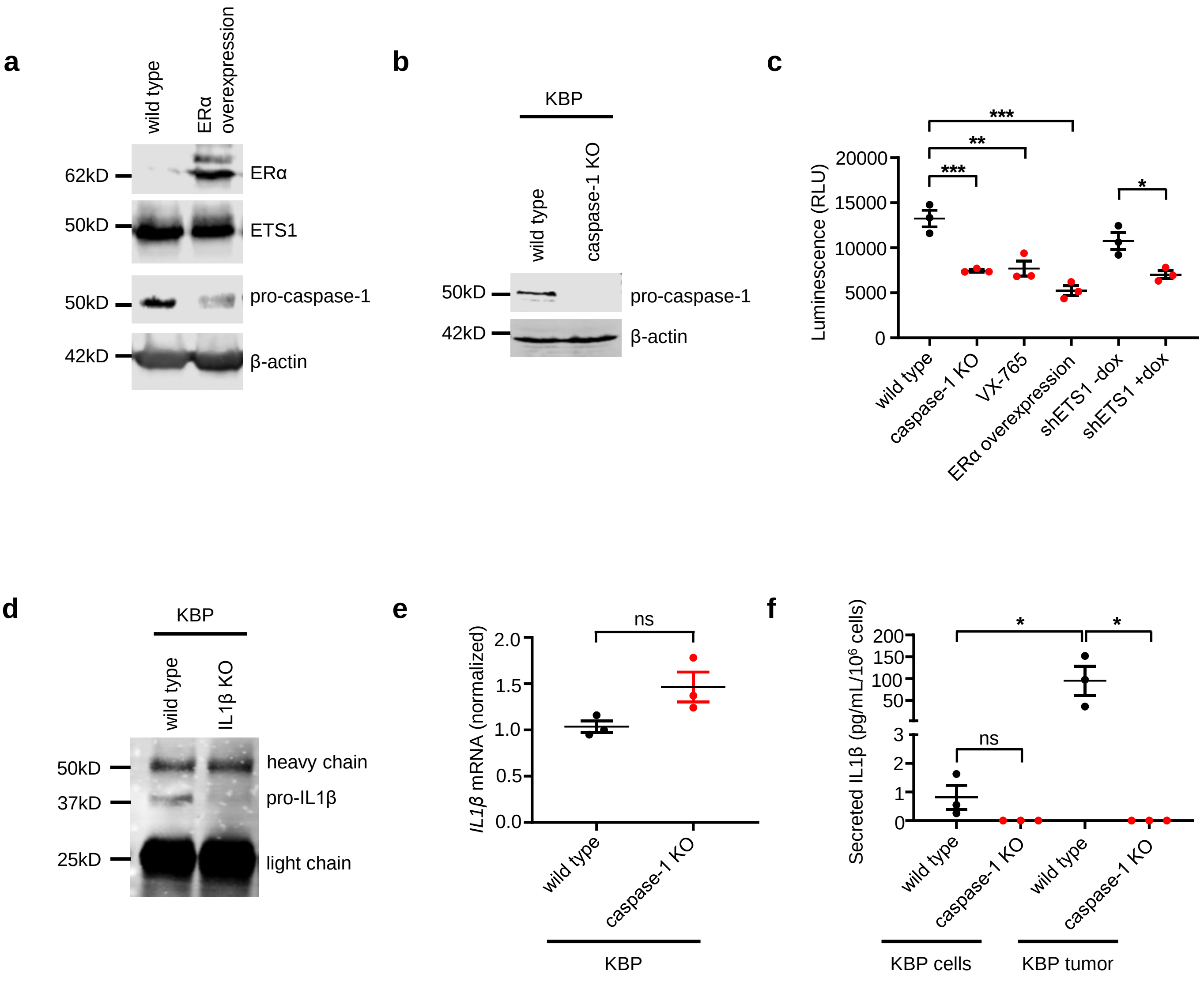

a
wild type
ERα overexpression
62kD
ERα
50kD
ETS1
50kD
pro-caspase-1
42kD
β-actin
b
KBP
wild type
caspase-1 KO
50kD
pro-caspase-1
42kD
β-actin
c
Luminescence (RLU)
20000
15000
10000
5000
0
***
**
***
*
wild type
caspase-1 KO
VX-765
ERα overexpression
shETS1 -dox
shETS1 +dox
d
KBP
wild type
IL1β KO
50kD
37kD
25kD
heavy chain
pro-IL1β
light chain
e
IL1β mRNA (normalized)
2.0
1.5
1.0
0.5
0.0
ns
wild type
caspase-1 KO
KBP
f
Secreted IL1β (pg/mL/10⁶ cells)
200
150
100
50
3
2
1
0
*
*
ns
wild type
caspase-1 KO
wild type
caspase-1 KO
KBP cells
KBP tumor

**Fig. S5. Caspase-1 production is ERα-, and ETS1-dependent, and is required for IL1β secretion in murine KBP cells. a** Immunoblot of ERα, ETS1, and pro-caspase-1 in wild type or ERα-overexpressing KBP cells. **b** Immunoblot of pro-caspase-1 in wild type and CRISPR-mediated caspase-1 KO KBP cells. β-actin is included as a loading control. **c** Analysis of caspase-1 activity (Caspase-1 Glo Inflammasome assay) in wild type, VX-765 (Belnacasan®) caspase-1 antagonist-treated, ERα-overexpressing and doxycycline-inducible shETS1 KBP cells. Mean with SEM, one-way ANOVA with Bonferroni's multiple comparisons test. * : p = 0.0393, ** : p = 0.0017, wild type vs caspase-1 KO: *** : p = 0.0009; wild type vs ERα overexpression: *** : p = 0.0006, n = 3. **d** Immunoprecipitation (anti-IL1β) immunoblot of IL1β in wild type and IL1β KO KBP cells. **e** IL1β mRNA levels detected by RTqPCR in wild type and caspase-1 KO KBP cells (n = 3 biologically independent experiments, mean with SEM, two-tailed, unpaired t-test with equal variances). **f** Secreted IL1β levels detected by ELISA in wild type and caspase-1 KO KBP cells in culture and harvested from KBP orthotopic tumors. One-way ANOVA with Bonferroni's multiple comparisons test for ELISA, ns = not significant, KBP cells vs tumor: * : p = 0.0245; KBP wild type vs caspase-1 KO tumor: * : p = 0.0233; n = 3. n = 3 biologically independent experiments for all immunoblots and representative images are shown. Source data are provided as a Source Data file.

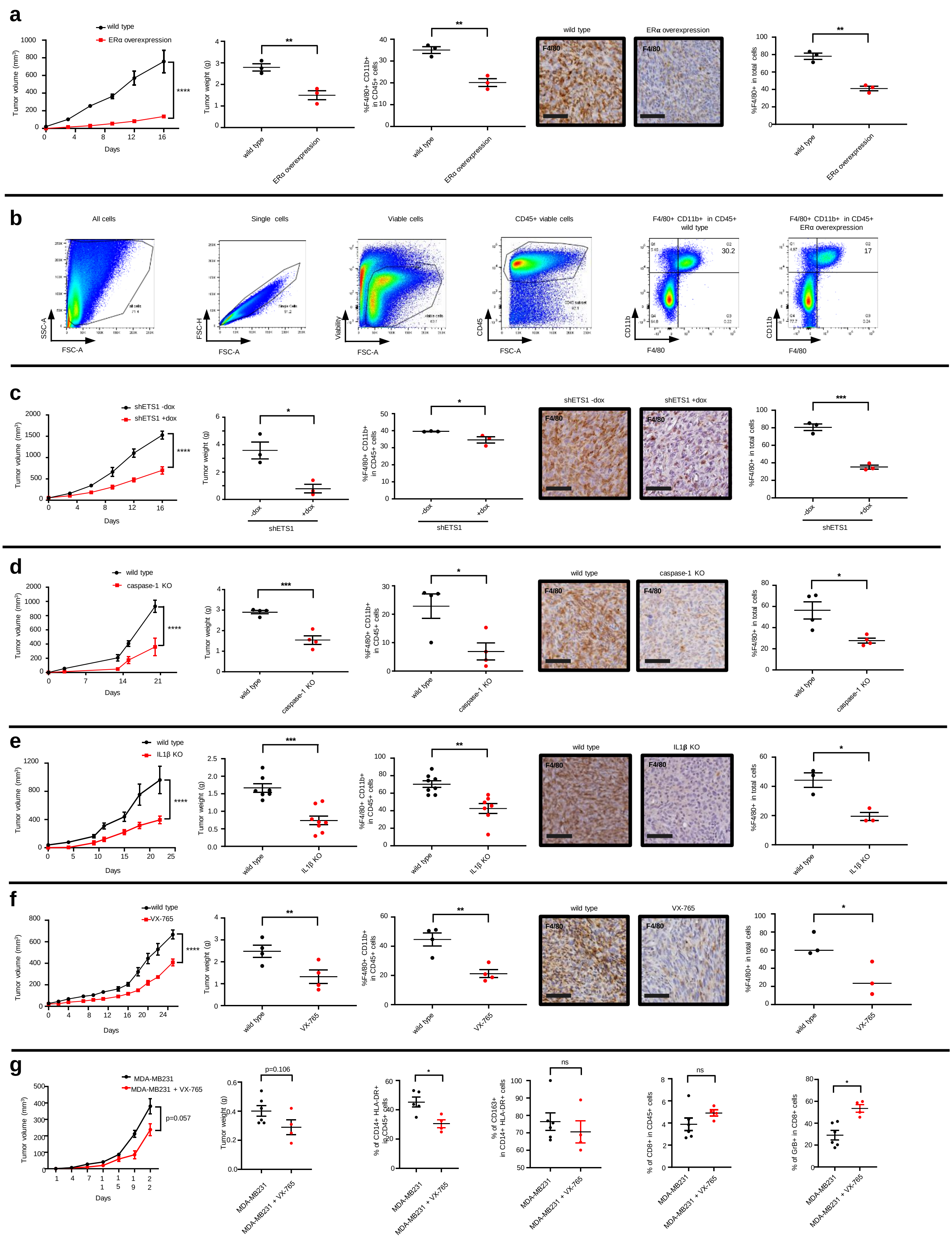
a
wild type
ERα overexpression
Tumor volume (mm³)
Days
****
Tumor weight (g)
**
%F4/80+ CD11b+ in CD45+ cells
F4/80
%F4/80+ in total cells
b
All cells
Single cells
Viable cells
CD45+ viable cells
F4/80+ CD11b+ in CD45+ wild type
F4/80+ CD11b+ in CD45+ ERα overexpression
SSC-A
FSC-A
FSC-H
Viability
CD45
CD11b
F4/80
30.2
17
c
shETS1 -dox
shETS1 +dox
-dox
+dox
shETS1
***
*
d
caspase-1 KO
e
IL1β KO
f
VX-765
g
MDA-MB231
MDA-MB231 + VX-765
p=0.057
p=0.106
ns
% of CD14+ HLA-DR+ in CD45+ cells
% of CD163+ in CD14+ HLA-DR+ cells
% of CD8+ in CD45+ cells
% of GrB+ in CD8+ cells

**Fig. S6. Inhibition of caspase-1 production mimics IL1β knockout and reduces macrophage infiltrates in mouse models of TNBC.** Growth curves, tumor weights, flow cytometric quantification of macrophages (F4/80 and CD11b double positive cells as a proportion of CD45 positive immune cells) and IHC staining/quantification of macrophages in the following TNBC mouse models: **a** Wild type and ERα-overexpressing KBP allografts (tumor weight: ** : $p = 0.0086$; F4/80+ CD11b+ in CD45+ cells: ** : $p = 0.0033$; F4/80+ in total cells: ** : $p = 0.0012$; **** : $p < 0.0001$; $n = 3$). **b** Representative FACS plots demonstrating the gating strategy for F4/80+ CD11b+ cells in CD45+ cells, in wild type and ERα-overexpressing KBP allografts. **c** KBP shETS1 tumors in mice fed regular (-dox) or doxycycline-containing (+dox) diet at tumor onset (tumor weight: * : $p = 0.016$; F4/80+ CD11b+ in CD45+ cells: * : $p = 0.0498$; *** : $p = 0.0005$; **** : $p < 0.0001$; $n = 3$). **d** Wild type and caspase-1 knockout (KO) KBP tumors (F4/80+ CD11b+ in CD45+ cells: * : $p = 0.024$; F4/80+ in total cells: * : $p = 0.0156$; *** : $p = 0.0009$; **** : $p < 0.0001$, $n = 4$). **e** Wild type and IL1β KO KBP tumors (* : $p = 0.0122$; ** : $p = 0.0011$; *** : $p = 0.0002$; **** : $p < 0.0001$, $n = 8$.). **f** Wild type KBP tumors treated with vehicle control or VX-765 (* : $p = 0.0414$; tumor weight: ** : $p = 0.0075$; F4/80+ CD11b+ in CD45+ cells: ** : $p = 0.0042$; **** : $p < 0.0001$. $n = 4$). **g** Growth curve, tumor weight, flow cytometric quantification of macrophages (CD14+ HLA-DR+, CD163+ M2-like) and T cells (CD8+ and GrB+) in humanized mice bearing MDA-MB231 xenografts, treated with vehicle control or VX-765 (CD14+ HLADR+ in CD45+ cells: * : $p = 0.0134$; %GrB+ in CD8+ cells: * : $p = 0.0227$, $n = 6$ ). Mean with SEM, two-way ANOVA with Bonferroni's multiple comparisons test for growth curves, two-tailed, unpaired t-test with equal variances for tumor weights and macrophage quantification. Scalebar corresponds to 50μm. Source data are provided as a Source Data file.

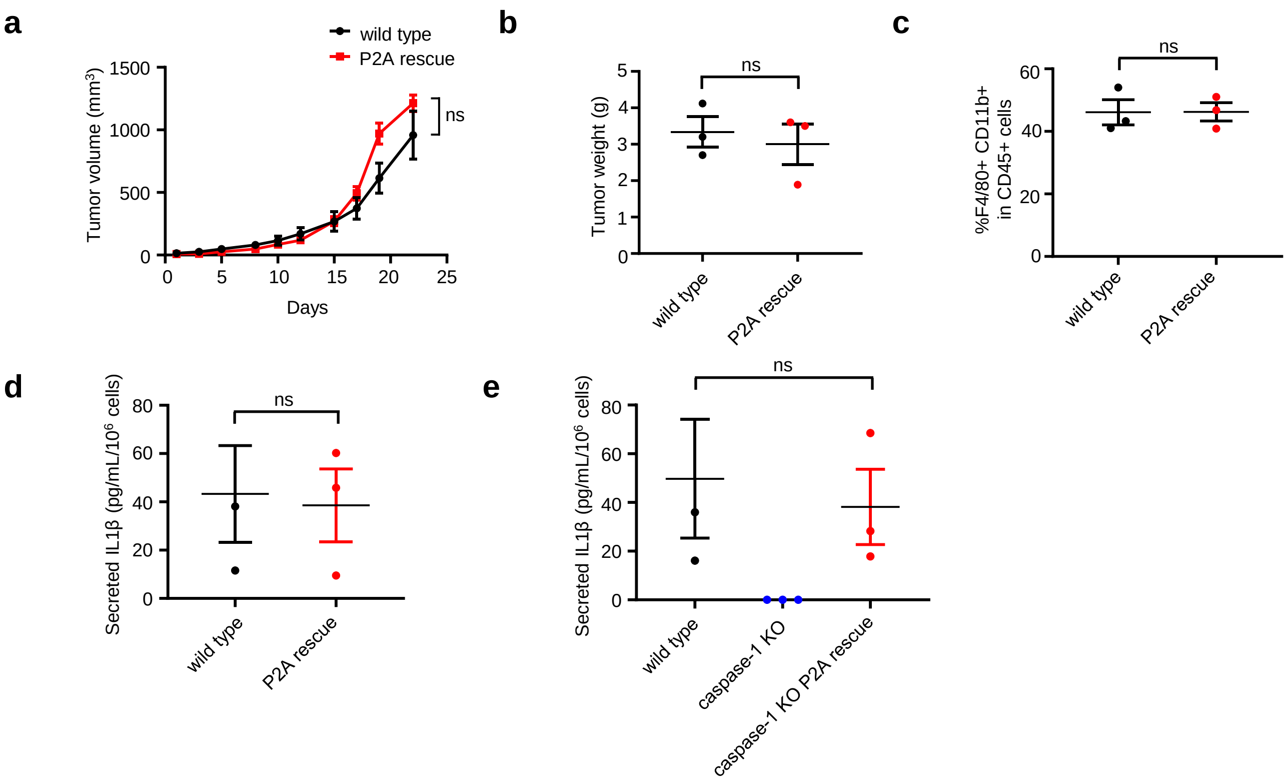
a
wild type
P2A rescue
Tumor volume (mm³)
Days
ns
b
Tumor weight (g)
ns
wild type
P2A rescue
c
%F4/80+ CD11b+ in CD45+ cells
ns
wild type
P2A rescue
d
Secreted IL1β (pg/mL/10⁶ cells)
ns
wild type
P2A rescue
e
Secreted IL1β (pg/mL/10⁶ cells)
ns
wild type
caspase-1 KO
caspase-1 KO P2A rescue

**Fig. S7. IL1β secretion, growth and macrophage recruitment of KBP *IL1β-P2A* variant tumors phenocopy wild type.** Growth curve (**a**), tumor weights at end point (n = 3) (**b**), and flow cytometry quantification of macrophages (F4/80 and CD11b double positive cells as a proportion of CD45 positive immune cells) (n = 3) (**c**) of wild type and P2A rescue KBP orthotopic tumors (n = 3). **d** ELISA measuring secreted IL1β from wild type and P2A rescue KBP orthotopic tumors (n = 3). **e** ELISA measuring secreted IL1β from wild type, caspase-1 KO, and caspase-1 KO P2A rescue KBP orthotopic tumors (n = 3). Mean with SEM, two-way ANOVA with Bonferroni's multiple comparisons test for growth curve, two-tailed, unpaired t-test with equal variances for tumor weight, macrophage quantification and ELISA, ns = not significant. Source data are provided as a Source Data file.

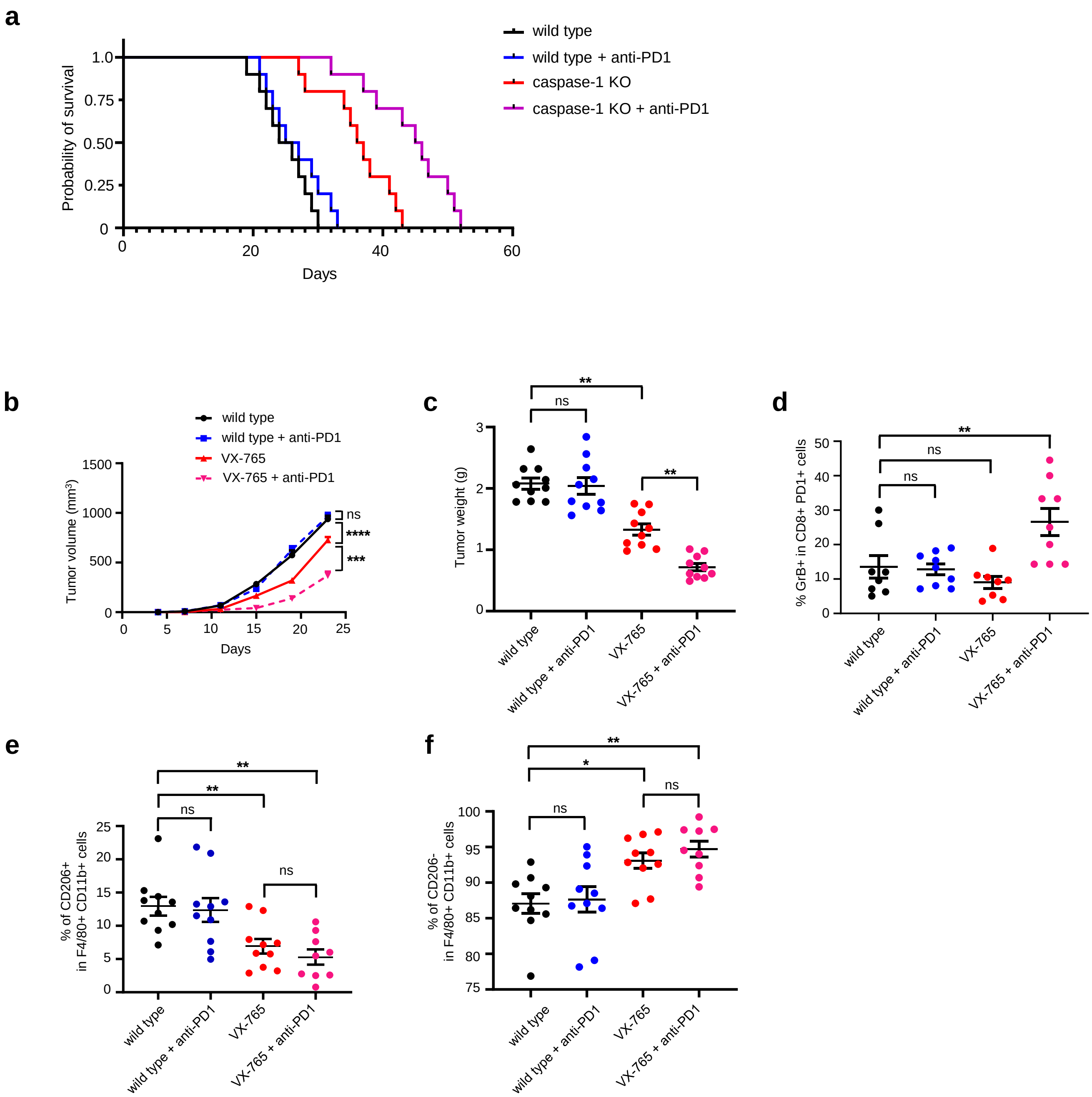
a
wild type
wild type + anti-PD1
caspase-1 KO
caspase-1 KO + anti-PD1
Probability of survival
1.0
0.75
0.50
0.25
0
0
20
40
60
Days
b
wild type
wild type + anti-PD1
VX-765
VX-765 + anti-PD1
Tumor volume (mm³)
1500
1000
500
0
0
5
10
15
20
25
Days
ns
****
***
c
Tumor weight (g)
3
2
1
0
**
ns
**
wild type
wild type + anti-PD1
VX-765
VX-765 + anti-PD1
d
% GrB+ in CD8+ PD1+ cells
50
40
30
20
10
0
**
ns
ns
wild type
wild type + anti-PD1
VX-765
VX-765 + anti-PD1
e
% of CD206+
in F4/80+ CD11b+ cells
25
20
15
10
5
0
**
**
ns
ns
wild type
wild type + anti-PD1
VX-765
VX-765 + anti-PD1
f
% of CD206-
in F4/80+ CD11b+ cells
100
95
90
85
80
75
**
*
ns
ns
wild type
wild type + anti-PD1
VX-765
VX-765 + anti-PD1

**Fig. S8. Caspase-1 neutralization improves the effect of anti-PD1 treatment in 4T1 and KBP TNBC mouse models. a** Kaplan-Meier survival curves of wild type and caspase-1 KO KBP orthotopic tumors treated with IgG control or anti-PD1 antibody. A log-rank test was used to determine the differences between wild type and wild type + anti-PD1; wild type and caspase-1 KO; wild type and caspase-1 KO + anti-PD1; caspase-1 KO and caspase-1 KO + anti-PD1 groups ($p = 0.24$, $p < 0.0001$, $p <0.0001$, and $p = 0.0023$, respectively, n = 10). Growth curve (*** : $p = 0.0002$; **** : $p < 0.0001$) (**b**), tumor weight (wild type vs VX-765: ** : $p = 0.002$; VX-765 vs VX-765 + anti-PD1: ** : $p = 0.002$, n = 10) (**c**), flow cytometry quantification of GrB+ CD8+ PD1+ T cells (** : $p = 0.0071$) (**d**), F4/80+ CD11b+ CD206+ macrophages (wild type vs VX-765: ** : $p = 0.0031$; wild type vs VX-765 + anti-PD1: ** : $p = 0.0086$, n = 10) (**e**), and F4/80+ CD11b+ CD206- macrophages (* : $p = 0.0187$; ** : $p = 0.0027$, n = 10) (**f**) in wild type and VX-765-treated 4T1 orthotopic tumors treated with IgG control or anti-PD1 antibody. Mean with SEM, two-way ANOVA with Tukey's multiple comparisons test for growth curves, one-way ANOVA with Tukey's multiple comparisons test for tumor weights and flow cytometry. ns = not significant. Source data are provided as a Source Data file.

**Table S1. Sequences of primers, siRNAs, shRNAs, and sgRNAs.**

| RTqPCR | Assay ID |
|---|---|
| human caspase-1 | Hs.PT.56a.22997425.g |
| human ERα | Hs.PT.58.14846478 |
| human ETS1 | Hs.PT.58.39917763 |
| human β-actin | Hs.PT.39a.22214847 |
| mouse caspase-1 | Mm.PT.58.12299102.g |
| mouse ERα | Mm.PT.58.8025728 |
| mouse IL1β | Mm.PT.58.41616450 |
| human β-actin | Mm.PT.58.28904620.g |
| ChIP assay | |
| human caspase-1 promoter | Forward primer: CGACATTCTCATTCCAGAGCCTATG |
| | Reverse primer: GACAGGGTCTCCTTGTGTTTCCTA |
| Cloning | |
| human ERα overexpressing plasmid | Forward primer: CGCGGATCCATGACCATGACCCTCCACAC |
| | Reverse primer: CCGGAATTCTCAGACCGTGGCAGGGAAAC |
| human ETS1 overexpressing plasmid | **Forward primer: CGCGGATCCATGCCAACTTTGTACAAAAA** |
| | **Reverse primer: CCGGAATTCTCACTCGTCGGCATCTGGCT** |
| mouse ERα overexpressing plasmid | **Forward primer: AATCCTGAATTCATGACCATGACCCTTCACAC** |
| | **Reverse primer: AATAGGATCCTCAGATCGTGTTGGGGAAGC** |
| caspase-1 CRISPR plasmid | Forward primer: CACCGAGGGCAAGACGTGTACGAG |
| | Reverse primer: AAACCTCGTACACGTCTTGCCCTC |

**Table S1 cont'd.**

| siRNA | Sequence |
|---|---|
| siscr | UGGUUUACAUGUCGACUAA<br>UGGUUUACAUGUUGUGUGA<br>UGGUUUACAUGUUUUCUGA<br>UGGUUUACAUGUUUUCCUA |
| siERα | GAUCAAACGCUCUAAGAAG<br>GAAUGUGCCUGGCUAGAGA<br>GAUGAAAGGUGGGAUACGA<br>GCCAGCAGGUGCCCUACUA |
| siETS1 | AUAGAGAGCUACGAUAGUU<br>GAAAUGAUGUCUCAAGCAU<br>GUGAAACCAUAUCAAGUUA<br>CAGAAUGACUACUUUGCUA |
| shRNA | |
| caspase-1 | ACACGTCTTGCTCTCATTA |
| ETS1 | AGGTGTAACAGGATTCTGG |
| CRISPR sgRNA | |
| caspase-1 (cloning method) | CTCGTACACGTCTTGCCCTC |
| caspase-1 (Synthego method) | UUAAACAGACAAGAUCCUGA |
| IL1β | CCAUUAGACAACUGCCAC |
| CRISPR ssODN | |
| P2A rescue<br>(red = P2A sequence) | TGATAACCTGCTGGTGTGTGACGTTCCCATTAGACAACTGCAC<br>GGCAGCGGAGCTACTAACTTCAGCCTGCTGAAGCAGGCTGGAGA<br>CGTGGAGGAGAACCCTGGACCTGTTCCCATTAGACAACTGCACTA<br>CAGACTCCGAGATGAACAACAAAAAAGCCTCGTGCTGTCGGA |

**Table S2. Antibodies.**

| Immunoblot | Source | Catalog no. | Working dilutions |
|---|---|---|---|
| Primary | | | |
| ERα | Cell Signaling | 8644S | 1: 500 |
| ETS1 | Cell Signaling | 14096S | 1: 1000 |
| caspase-1 | Cell Signaling | 3866S | 1: 500 |
| IL1 β | Cell Signaling | 83186 | 1: 200 |
| IL18 | Abcam | Ab68435 | 1: 500 |
| β-actin | Sigma | A5441 | 1: 2000 |
| mouse caspase-1 | Invitrogen | 14-9832-82 | 1: 500 |
| mouse IL1β | R&D Systems | AF-401-NA | 1: 200 |
| Secondary | | | |
| Donkey anti-rabbit-800CW | Mandel Scientific | 926-32213 | 1: 2000 |
| Donkey anti-goat-800CW | Mandel Scientific | 926-32214 | 1: 2000 |
| Goat anti-rat-800CW | Mandel Scientific | 926-32219 | 1: 2000 |
| Donkey anti-mouse-800CW | Mandel Scientific | 923-33212 | 1: 2000 |
| Goat anti-mouse IgG2a-680LT | Mandel Scientific | 926-68051 | 1: 2000 |
| Donkey anti-rabbit-680LT | Mandel Scientific | 926-68023 | 1: 2000 |
| Donkey anti-goat-680LT | Mandel Scientific | 926-68024 | 1: 2000 |
| Donkey anti-mouse-680LT | Mandel Scientific | 926-68022 | 1: 2000 |
| ChIP assay | | | |
| Isotype control rabbit monoclonal IgG | Cell Signaling | 3900S | 1: 200 |
| ETS1 | Cell Signaling | 14069S | 1: 200 |
| IL1β immunoprecipitation | | | |
| IL1β | R&D Systems | AF-401-NA | 1: 200 |
| IHC | | | |
| Primary | | | |
| F4/80 | ThermoFisher | MF48000 | 1: 400 |
| IL1 β | Abcam | ab9722 | 1: 400 |
| Pan-CK | Abcam | ab86734 | 1: 1000 |
| Secondary | | | |
| Rabbit IgG Biotinylated | Vector Laboratories | AK-5001 | 1: 1000 |
| Mouse IgG Peroxidase | Vector Laboratories | PK-4002 | 1: 1000 |
| Rat IgG Peroxidase | Vector Laboratories | PK-4004 | 1: 1000 |
| ELISA | | | |
| IL1β | R&D Systems | DY401-05 | |
| Flow cytometry | | | |
| viability | eBioscience | 65-0865-18 | 1: 1000 |
| mCD45 | eBioscience | 63-0451-82 | 1: 100 |
| mCD19 | eBioscience | 67-0193-82 | 1: 100 |

**Table S2 cont'd**

| Flow cytometry | | | |
|---|---|---|---|
| mF4/80 | BioLegend | 123128 | 1: 100 |
| mCD11b | BioLegend | 101212 | 1: 100 |
| mCD3 | BioLegend | 100234 | 1: 100 |
| mCD8 | eBioscience | 78-0081-82 | 1: 100 |
| mPD1 | eBioscience | 78-9985-82 | 1: 100 |
| mGranzyme B | eBioscience | 48-8898-82 | 1: 100 |
| mCD206 | ebioscience | 25-2601-80 | 1: 100 |
| hCD45 | Biolegend | 304048 | 1: 100 |
| hCD14 | Invitrogen | 12-0149-42 | 1: 100 |
| hHLA-DR | Biolegend | 307644 | 1: 100 |
| hCD163 | Biolegend | 333618 | 1: 100 |
| hCD8 | Invitrogen | 25-0087-42 | 1: 100 |
| hGranzyme B | Invitrogen | MHGB04 | 1: 100 |

| Multiplex IHC | Source | Catalog no. | Working dilutions |
|---|---|---|---|
| panCK | Agilent | M351529-2 | 1: 200 |
| caspase-1 | EMD Millipore | 06-503 | 1: 200 |
| CD3 | Roche | 790-4341 | 1: 200 |
| CD8 | Roche | 790-4460 | 1: 200 |
| CD68 | Agilent | M0814 | 1: 200 |
| CD163 | Biocare Medical | CM353M | 1: 200 |

**Table S3. Clinical details of patient samples obtained from the UHN Biobank**

IDC = invasive ductal carcinoma, All patient samples were obtained from surgical specimens.

| Sample IDs | Age at diagnosis | Breast cancer type | ER status |
|---|---|---|---|
| 63015 | 50-55 | IDC | Negative |
| 63082 | 60-65 | IDC | Negative |
| 64231 | 50-55 | IDC | Negative |
| 65044 | 80-85 | IDC | Negative |
| 66719 | 40-45 | IDC | Negative |
| 68316 | 75-80 | IDC | Negative |
| 69342 | 60-65 | IDC | Negative |
| 69489 | 40-45 | IDC | Negative |
| 71307 | 70-75 | IDC | Negative |
| 1036564 | 40-45 | IDC | Negative |
| 1054120 | 55-60 | IDC | Negative |
| 1095672 | 60-65 | IDC | Negative |
| 60195 | 70-75 | IDC | Positive |
| 62343 | 65-70 | IDC | Positive |
| 63080 | 40-45 | IDC | Positive |
| 64313 | 80-85 | IDC | Positive |
| 65811 | 70-75 | IDC | Positive |
| 66475 | 60-65 | IDC | Positive |
| 66501 | 60-65 | IDC | Positive |
| 67901 | 70-75 | IDC | Positive |
| 69739 | 50-55 | IDC | Positive |
| 919853 | 45-50 | IDC | Positive |
| 1368383 | 55-60 | IDC | Positive |
| 1441649 | 55-60 | IDC | Positive |

## Supplementary References


1 Cerami, E. *et al.* The cBio cancer genomics portal: an open platform for exploring multidimensional cancer genomics data. *Cancer Discov* **2**, 401-404, doi:10.1158/2159-8290.CD-12-0095 (2012). https://doi.org:10.1158/2159-8290.CD-12-0095

2 Gao, J. *et al.* Integrative analysis of complex cancer genomics and clinical profiles using the cBioPortal. *Sci Signal* **6**, pl1, doi:10.1126/scisignal.2004088 (2013). https://doi.org:10.1126/scisignal.2004088

3 Charafe-Jauffret, E. *et al.* Gene expression profiling of breast cell lines identifies potential new basal markers. *Oncogene* **25**, 2273-2284, doi:10.1038/sj.onc.1209254 (2006). https://doi.org:10.1038/sj.onc.1209254

4 Uhlen, M. *et al.* A human protein atlas for normal and cancer tissues based on antibody proteomics. *Mol Cell Proteomics* **4**, 1920-1932, doi:10.1074/mcp.M500279-MCP200 (2005). https://doi.org:10.1074/mcp.M500279-MCP200
