# Supplementary material for: Caspase-1-dependent spatiality in triple-negative breast cancer and response to immunotherapy
Source: Nat Commun. 2024 Oct 1;15:8514. doi: 10.1038/s41467-024-52553-6 (PMC11445480; doi:10.1038/s41467-024-52553-6)
Supplement: Supplementary file 3 — Reporting Summary [file 41467_2024_52553_MOESM3_ESM.pdf]

nature portfolio

Corresponding author(s): Michael Reedijk
Last updated by author(s): Sep 2, 2024

Reporting Summary

Nature Portfolio wishes to improve the reproducibility of the work that we publish. This form provides structure for consistency and transparency in reporting. For further information on Nature Portfolio policies, see our Editorial Policies and the Editorial Policy Checklist.

Statistics

For all statistical analyses, confirm that the following items are present in the figure legend, table legend, main text, or Methods section.

n/a	Confirmed	
☐	☒	The exact sample size (*n*) for each experimental group/condition, given as a discrete number and unit of measurement
☐	☒	A statement on whether measurements were taken from distinct samples or whether the same sample was measured repeatedly
☐	☒	The statistical test(s) used AND whether they are one- or two-sided<br>*Only common tests should be described solely by name; describe more complex techniques in the Methods section.*
☒	☐	A description of all covariates tested
☐	☒	A description of any assumptions or corrections, such as tests of normality and adjustment for multiple comparisons
☐	☒	A full description of the statistical parameters including central tendency (e.g. means) or other basic estimates (e.g. regression coefficient) AND variation (e.g. standard deviation) or associated estimates of uncertainty (e.g. confidence intervals)
☐	☒	For null hypothesis testing, the test statistic (e.g. *F*, *t*, *r*) with confidence intervals, effect sizes, degrees of freedom and *P* value noted<br>*Give P values as exact values whenever suitable.*
☒	☐	For Bayesian analysis, information on the choice of priors and Markov chain Monte Carlo settings
☒	☐	For hierarchical and complex designs, identification of the appropriate level for tests and full reporting of outcomes
☐	☒	Estimates of effect sizes (e.g. Cohen's *d*, Pearson's *r*), indicating how they were calculated

Our web collection on statistics for biologists contains articles on many of the points above.

Software and code

Policy information about availability of computer code

Data collection	Data collection for flow cytometry experiments was performed with BD FACSDiva software (v8.0.1). Data collection from the 7900HT fast real-time PCR system (Applied Biosystems) was performed using SDS (v2.4.1) real-time PCR software. Data collection from multicolor IHC was performed with inForm Tissue Analysis Software (Akoya Biosciences). Data collection from IHC staining was performed with Nanozoomer 2.0 HT (Hamamtsu).
Data analysis	FlowJo (v10.8.1), GraphPad Prism (v9.4.1), QuPath (v0.3.0), inForm Tissue Analysis Software (Akoya Biosciences)

For manuscripts utilizing custom algorithms or software that are central to the research but not yet described in published literature, software must be made available to editors and reviewers. We strongly encourage code deposition in a community repository (e.g. GitHub). See the Nature Portfolio guidelines for submitting code & software for further information.

Data

Policy information about availability of data

All manuscripts must include a data availability statement. This statement should provide the following information, where applicable:

- Accession codes, unique identifiers, or web links for publicly available datasets
- A description of any restrictions on data availability
- For clinical datasets or third party data, please ensure that the statement adheres to our policy

Microarray gene expression data from human breast cancer cell lines was downloaded from Charafe-Jauffret et al: doi: 10.1038/sj.onc.1209254. Human breast cancer tumor microarray (METABRIC) and RNAseq (TCGA PanCancer Atlas) gene expression and outcome data was obtained from cBioPortal: https://

www.cbioportal.org/

Human research participants

Policy information about studies involving human research participants and Sex and Gender in Research.

Reporting on sex and gender	*Use the terms sex (biological attribute) and gender (shaped by social and cultural circumstances) carefully in order to avoid confusing both terms. Indicate if findings apply to only one sex or gender; describe whether sex and gender were considered in study design whether sex and/or gender was determined based on self-reporting or assigned and methods used. Provide in the source data disaggregated sex and gender data where this information has been collected, and consent has been obtained for sharing of individual-level data; provide overall numbers in this Reporting Summary. Please state if this information has not been collected. Report sex- and gender-based analyses where performed, justify reasons for lack of sex- and gender-based analysis.*
Population characteristics	Formalin fixed paraffin-embedded (FFPE) human mammary tumor sections (Table S3) were obtained from the University Health Network (UHN) BioBank and analyzed by a pathologist for adequate quality
Recruitment	Tumors were collected by University Health Network biobank.
Ethics oversight	University Health Network Research Ethics Board has reviewed and approved the REB protocol 17-5109.

Note that full information on the approval of the study protocol must also be provided in the manuscript.

Field-specific reporting

Please select the one below that is the best fit for your research. If you are not sure, read the appropriate sections before making your selection.

- [x] Life sciences
- [ ] Behavioural & social sciences
- [ ] Ecological, evolutionary & environmental sciences

For a reference copy of the document with all sections, see nature.com/documents/nr-reporting-summary-flat.pdf

Life sciences study design

All studies must disclose on these points even when the disclosure is negative.

Sample size	Each group contained minimum 3 samples to reach statistical analysis significance.
Data exclusions	We did not exclude any data from our analyses.
Replication	To verify the reproducibility of our findings, experiments were performed using at least three biological replicates. All attempts at replication were successful.
Randomization	For the in vivo experiments, mice were randomly allocation to experimental groups once the tumor was palpable.
Blinding	The investigators were blinded to group allocation during data analysis.

Reporting for specific materials, systems and methods

We require information from authors about some types of materials, experimental systems and methods used in many studies. Here, indicate whether each material, system or method listed is relevant to your study. If you are not sure if a list item applies to your research, read the appropriate section before selecting a response.

Materials & experimental systems

n/a	Involved in the study	
☐	☒	Antibodies
☐	☒	Eukaryotic cell lines
☒	☐	Palaeontology and archaeology
☐	☒	Animals and other organisms
☐	☒	Clinical data
☒	☐	Dual use research of concern

Methods

n/a	Involved in the study	
☒	☐	ChIP-seq
☐	☒	Flow cytometry
☒	☐	MRI-based neuroimaging

Antibodies

Antibodies used

The following commercially available antibodies were used. Supplier's name and catalog number were provided.

From Cell Signaling Technology (Danvers, MA, USA):
1. ETS1 (#14096S)
2. caspase-1 (#3866S)
3. IL1β (#83186)

From Sigma-Aldrich, Inc (St Louis, MO, USA):
1. β-actin (#A5441)

From Invitrogen (Thermo Fisher Scientific, Waltham, MA, USA):
1. mouse caspase-1 (#14-9832-82)
2. hCD14 (#12-0149-42)
3. hCD8 (#25-0087-42)
4. hGranzyme B (MHGB04)

From R&D system (Minneapolis, MA, USA)
1. mouse IL1β (#AF-401-NA)

From Thermo Fisher Scientific (Waltham, MA, USA)
1. F4/80 (#MF48000)

From eBioscience (Thermo Fisher Scientific, Waltham, MA, USA):
1. mCD45 (#63-0451-82)
2. mCD19 (#67-0193-82)
3. mCD8 (#78-0081-82)
4. mPD1 (#78-9985-82)
5. mGranzyme B (#48-8898-82)
6. mCD206 (#25-2601-80)

From BioLegend (San Diego, CA, USA):
1. mF4/80 (#123128)
2. mCD11b (#101212)
3. mCD3 (#100234)
4. hCD45 (#304048)
5. hHLA-DR (#307644)

From EMD Millipore (Burlington, MA, USA):
1. caspase-1 (#06-503)

From Roche Diagnostics (Basel, Switzerland):
1. CD3 (#790-4341)
2. CD8 (#790-4460)

From Agilent Technologies, Inc (Santa Clara, CA, USA):
1. panCK (#M351529-2)
2. CD68 (#M0814)

From Biocare Medical (Pacheco, CA, USA):
1. CD163 (#CM353M)

From Abcam (Cambridge, UK):
1. IL1β (ab9722)
2. panCK (ab86734)

Validation

Validation statements for all antibodies listed above can be found through the following links to the manufacturer's website.

From Cell Signaling Technology (Danvers, MA, USA):
1. https://www.cellsignal.com/products/primary-antibodies/ets-1-d8o8a-rabbit-mab/14069
2. https://www.cellsignal.com/products/primary-antibodies/caspase-1-d7f10-rabbit-mab/3866
3. https://www.cellsignal.com/products/primary-antibodies/cleaved-il-1b-asp116-d3a3z-rabbit-mab/83186

From Sigma-Aldrich, Inc (St Louis, MO, USA):
1. https://www.sigmaaldrich.com/CA/en/product/sigma/a5441

From Invitrogen (Thermo Fisher Scientific, Waltham, MA, USA):
1. https://www.thermofisher.com/antibody/product/Caspase-1-Antibody-clone-5B10-Monoclonal/14-9832-82

2. https://www.thermofisher.com/antibody/product/CD14-Antibody-clone-61D3-Monoclonal/12-0149-42
3. https://www.thermofisher.com/antibody/product/CD8a-Antibody-clone-SK1-Monoclonal/25-0087-42
4. https://www.thermofisher.com/antibody/product/Granzyme-B-Antibody-clone-GB12-Monoclonal/MHGB04

From R&D system (Minneapolis, MA, USA):
1. https://www.rndsystems.com/products/mouse-il-1beta-il-1f2-antibody_af-401-na

From Thermo Fisher Scientific (Waltham, MA, USA):
1. https://www.thermofisher.com/antibody/product/F4-80-Antibody-clone-BM8-Monoclonal/MF48000

From eBioscience (Thermo Fisher Scientific, Waltham, MA, USA):
1. https://www.thermofisher.com/antibody/product/CD45-Antibody-clone-30-F11-Monoclonal/63-0451-82
2. https://www.thermofisher.com/antibody/product/CD19-Antibody-clone-eBio1D3-1D3-Monoclonal/67-0193-82
3. https://www.thermofisher.com/antibody/product/CD8a-Antibody-clone-53-6-7-Monoclonal/78-0081-82
4. https://www.thermofisher.com/antibody/product/CD279-PD-1-Antibody-clone-J43-Monoclonal/78-9985-82
5. https://www.thermofisher.com/antibody/product/Granzyme-B-Antibody-clone-NGZB-Monoclonal/48-8898-82
6. https://www.thermofisher.com/antibody/product/CD200R3-Antibody-clone-Ba13-Monoclonal/25-2001-80

From BioLegend (San Diego, CA, USA):
1. https://www.biolegend.com/de-de/products/percp-cyanine5-5-anti-mouse-f480-antibody-4303
2. https://www.biolegend.com/de-de/products/apc-anti-mouse-human-cd11b-antibody-345
3. https://www.biolegend.com/de-de/products/brilliant-violet-510-anti-mouse-cd3-antibody-7990
4. https://www.biolegend.com/en-ie/products/brilliant-violet-785-anti-human-cd45-antibody-9325
5. hhttps://www.biolegend.com/fr-ch/products/brilliant-violet-711-anti-human-hla-dr-antibody-7939

From EMD Millipore (Burlington, MA, USA):
1. https://www.emdmillipore.com/US/en/product/Anti-Caspase-1-Antibody,MM_NF-06-503

From Roche Diagnostics (Basel, Switzerland):
1. https://diagnostics.roche.com/global/en/products/lab/cd3-2gv6-confirm-rtd000768.html
2. https://diagnostics.roche.com/global/en/products/lab/cd8-sp57-confirm-rtd000777.html

From Agilent Technologies, Inc (Santa Clara, CA, USA):
1. https://www.agilent.com/store/productDetail.jsp?catalogId=M351529-2
2. https://www.agilent.com/store/productDetail.jsp?catalogId=M081401-2

From Biocare Medical (Pacheco, CA, USA):
1. https://biocare.net/product/cd163-antibody/

From Abcam (Cambridge, UK):
1. https://www.abcam.com/en-ca/products/primary-antibodies/il-1-beta-antibody-ab9722
2. https://www.abcam.com/en-ca/products/primary-antibodies/pan-cytokeratin-antibody-ae1-ae3-5d3-ab86734

Eukaryotic cell lines

Policy information about cell lines and Sex and Gender in Research

Cell line source(s)	Human breast cancer cell line MCF7, MD-MBA-231 were purchased from American Type Culture Collection (ATCC). KBP cells were obtained from Dr. Chiara Gorrini.
Authentication	MCF7 and MD-MBA-231 cell lines were authenticated through STR analysis.
Mycoplasma contamination	All cell lines tested negative for mycoplasma.
Commonly misidentified lines (See ICLAC register)	No commonly misidentified cell lines were used.

Animals and other research organisms

Policy information about studies involving animals; ARRIVE guidelines recommended for reporting animal research, and Sex and Gender in Research

Laboratory animals	6-12 week old female FVB mice and CD34+ Hu-NSG mice were purchased from The Jackson Laboratory. All animals were housed in the animal facility at the Princess Margaret Cancer Centre. Dark/light cycle, ambient temperature and humidity were centrally-regulated and animals were closely monitored by resident veterinarians for well-being.

Wild animals	The study did not involve wild animals.
Reporting on sex	Only female mice were used in the study.
Field-collected samples	The study did not involve samples collected from the field.
Ethics oversight	All animal experiments were performed with approval from the Animal Care Committee of University Health Network. Animals were euthanized when tumors reached 1.5 cm in diameter.

Note that full information on the approval of the study protocol must also be provided in the manuscript.

Clinical data

Policy information about clinical studies
All manuscripts should comply with the ICMJEguidelines for publication of clinical research and a completedCONSORT checklist must be included with all submissions.

Clinical trial registration	*Provide the trial registration number from ClinicalTrials.gov or an equivalent agency.*
Study protocol	*Note where the full trial protocol can be accessed OR if not available, explain why.*
Data collection	*Describe the settings and locales of data collection, noting the time periods of recruitment and data collection.*
Outcomes	*Describe how you pre-defined primary and secondary outcome measures and how you assessed these measures.*

Flow Cytometry

Plots

Confirm that:

- [x] The axis labels state the marker and fluorochrome used (e.g. CD4-FITC).
- [x] The axis scales are clearly visible. Include numbers along axes only for bottom left plot of group (a 'group' is an analysis of identical markers).
- [ ] All plots are contour plots with outliers or pseudocolor plots.
- [x] A numerical value for number of cells or percentage (with statistics) is provided.

Methodology

Sample preparation	Tumors were minced and incubated in digestion buffer [1 mg/mL of collagenase (Sigma) and 10 µg/mL of Pulmozyme (Roche), 2 mM L-glutamine (Lonza), 100 µg/mL P/S (Lonza) in Iscove's Modified Dulbecco's Medium (IMDM), Gibco] at 37C for 40-60min. The digested samples were filtered through a 70µm Falcon cell strainer, stained with a fixable viability dye and fluorophore-conjugated antibodies
Instrument	Cells were analyzed using BD Biosciences LSR Fortessa flow cytometer
Software	Data collection was performed using FACSDiva software (BD). Data was analyzed using FlowJo software (TreeStar, v10.8.1)
Cell population abundance	Tumors were analyzed by FACS and none of cell populations were sorted.
Gating strategy	The cells were first gated on viability dye and followed by specific antibodies as described in the main text. A logscale expression value of 10e3 was used as a threshold to gate each population.

- [ ] Tick this box to confirm that a figure exemplifying the gating strategy is provided in the Supplementary Information.
